# The military spouse experience of living alongside their serving/veteran partner with a mental health issue: A systematic review and narrative synthesis

**DOI:** 10.1371/journal.pone.0285714

**Published:** 2023-05-18

**Authors:** Emma Senior, Amanda Clarke, Gemma Wilson-Menzfeld

**Affiliations:** Department of Nursing, Midwifery and Health, Northumbria University, Newcastle-Upon-Tyne, United Kingdom; St John’s University, UNITED STATES

## Abstract

**Introduction:**

Military healthcare studies have reported a wide range of mental health issues amongst military personnel. Globally, mental health issues are one of the main causes of ill health. Military personnel have a greater prevalence of mental health issues than that of the general population. The impact of mental health issues can be wide and far reaching for family and carers. This systematic narrative review explores the military spouse experience of living alongside their serving or veteran partner with a mental health issue.

**Methods:**

The systematic review performed was based on the PRISMA guide for searching, screening, selecting papers for data extraction and evaluation. Studies were identified from CINHAL, ASSIA, Proquest Psychology, Proquest Nursing & Allied Health source, Proquest Dissertations & Theses, ETHOS, PsychArticles, Hospital collection, Medline, Science Direct Freedom Collection and hand searching of citations and reference lists.

**Results:**

Twenty-seven studies were included in the narrative synthesis. Five overarching themes from the experiences of military spouses’ living alongside their serving/veteran partners mental health issue were identified: caregiver burden, intimate relationships, psychological/psychosocial effects on the spouse, mental health service provision and spouse’s knowledge and management of symptoms.

**Conclusions:**

The systematic review and narrative synthesis identified that the majority of studies focused on spouses of veterans, very few were specific to serving military personnel, but similarities were noted. Findings suggest that care burden and a negative impact on the intimate relationship is evident, therefore highlight a need to support and protect the military spouse and their serving partner. Likewise, there is a need for greater knowledge, access and inclusion of the military spouse, in the care and treatment provision of their serving partner’s mental health issue.

## Introduction

Globally, mental health issues are one of the main causes of ill health, accounting for 13% of disease burden and, by 2030, this figure is predicted to rise to 15% [1, 2]. Worldwide, major depression is considered to be the second leading cause of disability, [3] with depression, anxiety and drug use reported as the primary drivers of disability in those aged between 20–29 years [4]. It is also estimated that a quarter of the population will at some point in their lives suffer from a mental health illness [5].

Military studies have reported a wide range of mental health issues with active serving personnel [6, 7] and veterans alike [8, 9]. Indicative of many roles within the military, is the exposure to combat and trauma [8, 9]. High combat exposure has been associated with a deterioration in mental health and an increased risk of suicide [8, 9]. Figures suggest for military personnel the prevalence of depression is between 23–26% [6], considerably higher than the general population globally, where the figure is estimated at 13–15% [1]. It is also suggested that up to 60% of military personnel with a mental health issue will not seek treatment [10].

In general, a lack of healthcare management models, resulting from underfunding and austerity measures [11], has led to care provision for those with a mental health issue predominantly falling to family members [12]. The impact on the quality of life for family caregivers can be wide and far reaching [13]. Studies suggest a correlation between care burden and adverse health effects, such as increased stress, physical exhaustion, anxiety and depression for the caregiver [14].

From a military context, the adverse health effects of the caregiver are further compounded, because even without the existence of a mental health issue, it is recognised that there are significant effects on the military family unit, such as long separations, and 24 hour working patterns. This is especially pertinent during times of deployment with military families experiencing a higher prevalence of psychological disorders [6]. It has also been reported that the presence of a mental health issue is the second most leading cause of divorce within serving military populations [15]. Whilst there is a plethora of literature surrounding families and deployment [16, 17], a preliminary scope of the literature showed that little is known about the experience of military spouses living alongside serving partners with a mental health issue. This literature review aims to explore the experience of the military spouse during their serving partners mental health issue.

## Materials and methods

In acknowledgment of the limited evidence around the research topic found from the initial scope of the literature, a systematic review with narrative synthesis was executed to enable the inclusion of a wide range of literature and research designs [18]. Qualitative evidence can answer different but often complementary questions to quantitative evidence [18]. A systematic review assumes a narrative synthesis approach concerned with generating new insights and recommendations textually [18, 19]. Narrative synthesis brings together findings from all the included studies to capture conclusions. Using a deductive approach, these conclusions form thematic groups based on the body of evidence as a whole [18, 19]. The review did, however, follow the steps documented in the Preferred Reporting Items for Systematic review and Meta-Analysis (PRISMA) statement [20] **See** S1 File: **PRISMA checklist**.

**Fig 1:**
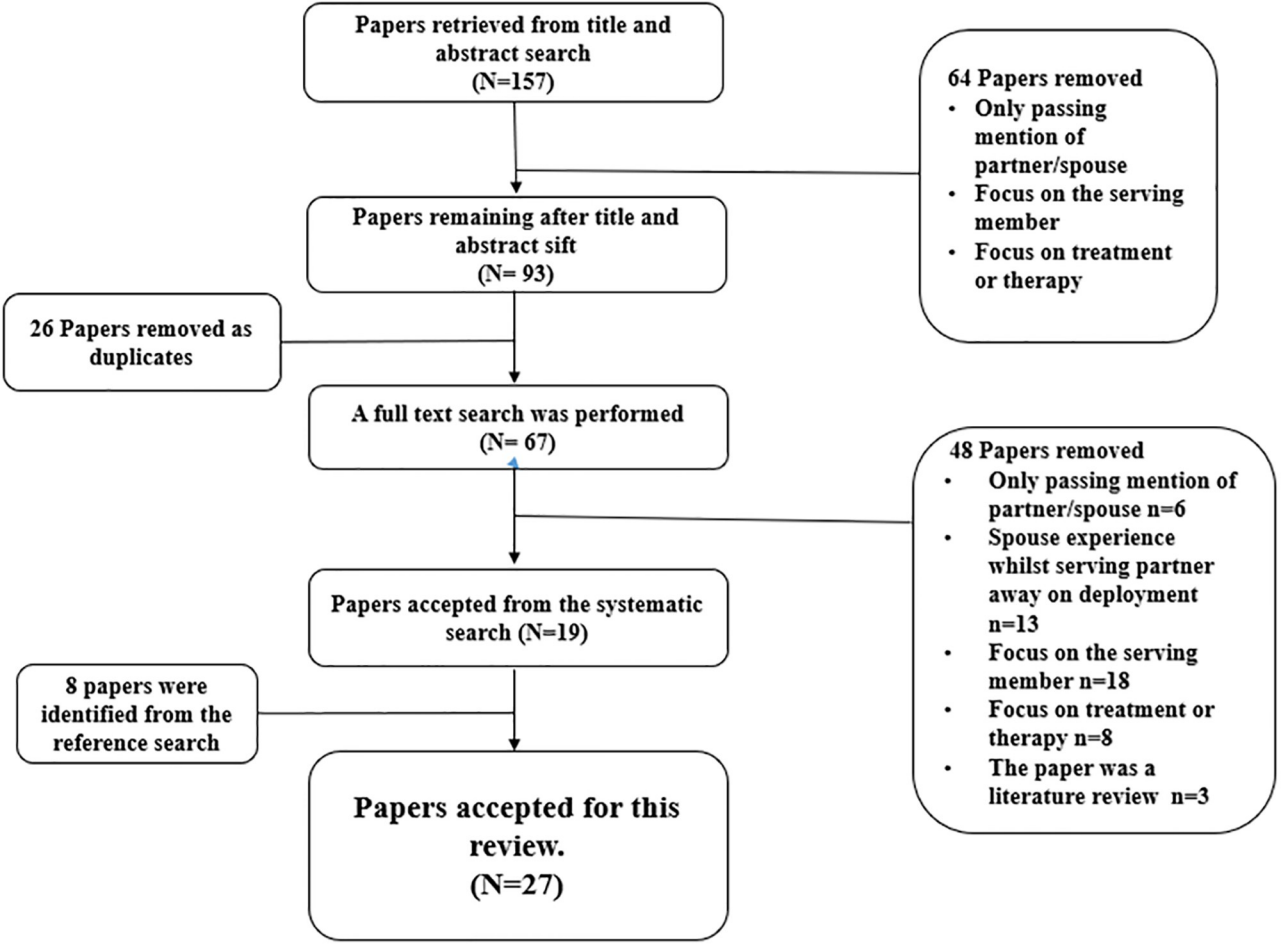
Systematic search strategy.

This review specifically focuses on the spouse experience, and only aims to include studies whereby the spouse is identified in the aim or outcome. From the research aim, search terms were developed using the framework PICO (Table 1) and a systematic search strategy (Fig 1) was utilised to ensure that the searches are comprehensive and transparent [18].

**Table 1:** PICO framework.

Search term development	
P—Patient or population	(Wife; husband; spouse; partner) AND (armed forces; Army; Navy; Airforce; military; soldier/s—truncated to soldi*, tri-service; sailor/s; airman/airmen; marine)
I—Intervention	Mental; psychological; psychology; psychologist (truncated to psychologi*)
C—Comparison (if applicable)	Not applicable
O—Outcome	Support; experience, care; caring; carer (truncated to car*)

Suitable databases were identified and used for the searches: CINHAL, ASSIA, Proquest Psychology, Proquest Nursing & Allied Health source, Proquest Dissertations & Theses, ETHOS, PsychArticles, Hospital collection, Medline, Science Direct Freedom Collection. All relevant search terms were utilised, and initial searches yielded limited literature specific to serving military spouses, so the parameters of the search were widened to include veteran spouses. The time parameter was also broadened to include any papers from any publication date; however, consideration was given only to those papers written in the English language. Owing to the cultural complexities, studies conducted with westernised military spouses published in peer reviewed English language journals were used. The searches were completed between July 2021 and March 2022. A total of one hundred and fifty-seven papers were retrieved from the initial searches with ninety-three being deemed of some relevance following a title and abstract sift (Fig 1).

Twenty six papers were removed as duplicates and following a full-text search, a further forty eight papers were excluded as: only passing mention of spouse (n = 6), the spouse experience was specific to during the time of deployment (n = 13), the focus of the study was the serving/veteran partner (n = 18), the focus was directed at a treatment or therapy (n = 8) and the remaining were literature reviews (n = 3). Reference and citation searches were executed on all relevant papers, resulting in eight further papers eligible for inclusion bringing the total to twenty-seven.

From the twenty-seven papers included in the review, the study aim, sample size, method and tools plus the location of study were extracted (Table 2). Steps 2–4 of the Economic Social Research Council’s (ESRC) guidance on the conduct of narrative synthesis was employed [19]. This guidance proposes four stages; however, the process is iterative, encouraging the researcher to move freely within each stage and not approach them linearly in a sequential manner [19]. Stage one was excluded since developing a theoretical model of how an intervention works and for whom was not an aim of the review [19]. In stage 2 and 3 the initial synthesis of the findings in the included papers was completed followed by an exploration of the relationship between the findings. Stage 4 required the research team to assess the quality of the synthesis.

**Table 2:** Overview of papers.

Author	Aim	Sample size	Method	
Allen, E.S, Rhodes, G.K. Stanley, S.M. & Markman, H.J. (2010) [25]	Toexamine whether a recent history of deployment and current PTSD symptoms are related to several aspects of marital functioning.	434 couples	Quantitative—questionnaire of self reported measures: Deployment PTSD Checklist (PCL) Relationship functioning Which included Kansas Marital Satisfaction Scale (KMS) Confidence Scale Positive Bonding Scale, Parenting Alliance Inventory (PAI) Dedication Scale Satisfaction with Sacrifice Scale Communications Danger Signs Scale	USA
Beckman, J.C, Lytle, B.L. & Feldman, M.E. (1996) [26]	To studyprospectively burden in partners of Vietnam veterans with PTSD—a) to examine changes in caregiver burden that occur over time b) to determine the degree to which caregiver burden relates to their own adjustment c) to evaluate the degree to which changes in caregiver burden relate to changes in adjustment over the follow-up	58 couples- Vietnam war veterans and partners	Quantitative—mail out survey comprising: demographic information, Burden Interview (BI), Symptom Checklist -90- Revised (SCL-90-R) Beck Depression Inventory (BDI) Speilberger State and Trait Anxiety Inventory (STAI)	USA
Brown, V. A. (2015) [27]	To gain an in-depth understanding or essence of the transformative experiences of partners of junior and mid-grade enlisted soldiers in the rank of E-1 to E-6 who were wounded in combat operations in support of the War, to learn how these women learn to make new meaning of their life circumstances as a result of profound and dramatic changes in their lives and life experiences as a result of those injuries and to determine how society can better support them.	15 women spouses (5 x face to face interviews and 10 x over telephone interviews)	Qualitative—Hermeneutic/interpretive phenomenological methodolgy. Data collected in two phases: Phase 1—participant profile questionnaires Phase 2—individual interviews utilising an individual interview protocol consisting of statements, definitions and questions to frame interviews.	USA
Buchanan, C. Kemppainen, J. Smith, S. MacKain, S. & Wilson, C. (2011) [28]	To identify perspectives of female spouses/intimate partners regarding posttraumatic stress disorder in returning Iraq and Afghanistan combat veterans	34 x participants	Mixed—Quantitative approach for demographic data Qualitative approach using critical interviews to explore with partners: How they would know if their spouse needed treatment for PTSD What behaviours would indicate to them that their spouse was willing to receive treatment for PTSD In the event that their spouse needed treatment and resisted it, what would it take on their part to get them into treatment.	USA
Campbell, S.B. & Renshaw, K.D. (2013) [29]	To examine the associations among a) service members‘PTSD symptoms b) service members‘ emotional disclosure to partners c) relationship satisfaction in both partners within military couples over two time points.	Time point 1 = 224 service members and 214 partners Time point 2 = 83 service members and 91 partners	Quantitative Time point 1—questionnaire given in person Time point 2- questionnaire emailed to participants Measures used: PTSD Checklist (PCL) Likelihood of Disclosure Scale Relationship Assessment Scale (RAS) To test three seperate hypothesis Association of service members PTSD symptoms at time 1, service person and partner report of disclosure at time 2 and service members and partners relationship satisfaction at time 2 Emotional numbing would be the individual cluster most strongly associated with relationship satisfaction for both partners That service members emotional disclosure at time 2 would mediate associations of service members time 1 PTSD symptoms with both partners time 2 relationship satisfaction.	USA
Daniels, J. A. (2013) [30]	To examine the relationship between the level of burden of care and the perception of social support reported by spouses of OIF and OEF reserve veterans who have been diagnosed with PTSD	61 x participants	Quantitative—survey monkey developed to include: Demographic questionnaire Zarit Brden Interview (ZBI) which used a 5 point Likert Scale to measure burden Social Support Index (SSI) To test the four seperate hypothesis; namely: The level of burden of care reported by the spouse of OIF and OEF reserve veterans diagnosed with PTSD will be negatively correlated with the level of perceived social support reported. The level of burden of care reported by the spouses of OIF and OEF reserve veterans diagnosed with PTSD will be higher when there are dependant children in the home. The level of perceived social support reported by the spouse of OIF and OEF reserve verterans diagnosed with PTSD will be lower when there are dependant children living in the home. The level of burden of care reported by spouses of OIE and OEF reserve verterans diagnosed with PTSD who have dependant children living in the home will be negatively correlated with the level of perceived social support.	USA
Iniedu, A. O. E. (2010) [31]	To assess the incidence of STS in and explore the experiences of a sample of wives of Iraq and Afghanistan war veterans diagnosed with PTSD.	10 x participants	Mixed methods -Quatitative assessment tools and interviews. Quantitative methods used Basic demographic qusetionnaire Secondary Traumatic Stress Scale (STSS) with a score of 38 or above to be considered diagnostic of STS Qualitative method used Face-to-face semi-structured interview	USA
Jordan, B.K, Marmar, C.R, Fairbank, J.A, Schlenger, W.E, Kulka, R.A, Hough, R.L. & Weiss, D.S (1992) [32]	To improve on the methodology of previous studies of the effects of PTSD on families by recruiting a community sample rather than a treatment seeking sample to compare levels of problems in the families of Vietnam veterans with or without PTSD	862 veterans & 376 spouse/partners	Quantitative—Interview using questionnaire. Not all measures included the spouse/partner. Indexes given to Spouse/Partner for completion were Marital Problem Index Family Adjustment Index Level of Life Functioning Index Family Violence Measures Index of Subjective Wellbeing PERI Demoralisation Scale Social Isolation Index Alcohol Problems Measure Drug Problems Measure Nervour Breakdown Measure Total Behaviour Problems Score from the Child Behaviour Checklist	USA
Lyons, M. A. (1999) [33]	To examine what is like for the wives/female partners to live with a Vietnam veteran who suffers from Post-traumatic stress disorder.	10 x participants	Qualitative—phenomenological study Interviews with open ended questions were used to ask the partners of veterans: What their early relationship was like What PTSD symptoms their partner experiences and how they cope with them What feelings they experience in their relationship with their partner Why they stay or why they leave How different they are now from the start of the relationship/before their partner experienced symptoms	USA
Manguno-Mire, G. Sautter, F. Lyons, J. Myers, L. Perry, D. Sherman, M. Glynn, S. & Sullivan, G. (2007) [34]	To examine the psychological distress among co-habiting female partners of combat veterans with posttraumatic stress disorder.	89 co-habiting partners	Quantitative—telephone survey using the Partner Experiences with PTSD survey (PEPS)—a synthesis of three published instruments: PTSD Checklist (Military) Burden Inventory Brief Symptom Inventory and seven study-specific scales developed by a team of content experts in PTSD treatment and family therapy	USA
Mansfield, A.J. Schaper, K.M. Yanagida, A.M. & Rosen, C.S. (2014) [35]	To provide a description of the lived experience of partners of veterans diagnosed with PTSD by examining the written words of their significant other.	252 x participants	Qualitative A smaller study using the results of a Quantitative—postal survey which included an option for written comments; namely: If there is anything else that you would like to share relating to PTSD in veterans and your own experiences, please do so below. The written comments were analysed qualitatively	USA
Renshaw, K.D. & Caska, C.M. (2012) [36]	To determine whether patterns of findings replicated across fairly large samples from two separate combat eras.	Study 1 = 258 couples in which one member had served in the Utah National Guard/ reserves during Operation Enduring freedom and 9 Iraqi freedom Study 2 = 465 couples who participated in the National Vietnam readjustment study	Quantitative—questionnaires Study 1: PTSD checklist (PCL) Adapted PCL for partners Depression Anxiety Stress Scale (DASS) Relationship Assessment Scale (RAS) Study 2: Mississippi Scale for Combat-Related PTSD Marital Problems Index (MPI) Psychological Distress Index (PDI)	USA
Riggs, D.S, Byrne, C.A., Weathers, F.W. & Litz, B.T. (1998) [37]	To examine the association of relationship difficulties with clusters of PTSD symptoms	50 couples—Vietnam veterans and their female partners	Quantitative—self-reporting questionnaires which included Dyadic Adjustment Scale (DAS) Marital Status Inventory (MSI) Relationship Problems Scale (RPS) Fear of Intimacy Scale (FIS) PTSD Checklist- Military version (PCL-M) All questionnaires used were to test PTSD and relationship distress PTSD and measures of relationship quality Association of relationship quality and PTSD symptom clusters Emotional numbing and effortful avoidance associated with relationship difficulties	USA
Sautter, F. Lyons, J.A. Manguno-Mire, G, Perry, D. Han, X. Sherman, M. Myers, L. Landis, R & Sullivan, G. (2006) [38]	To use the behavioural model of health service utilization to identify predictors of partner PTSD treatment engagement.	83 x participants	Quantitative—Telephone survey using: Patient PTSD Survey (PPS) Partner Experiences with PTSD Survey (PEP) The PEP scale measured the following: Patient-Partner Involvement Perceived Barriers to Treatment Perceived Benefits of Treatment PTSD Self-efficacy Caregiver Burden Partner PTSD Treatment Engagement.	USA
Sherman, M.D. Blevin, D. Kirchner, J Ridner, L & Jackson, T (2008) [39]	To a) explore the perceived appropriateness and potential benefits to partner participation, b) identify perceived risks and barriers to partner participation, c) use these findings to develop strategies to solicit partner involvement in mental health treatment.	10 couples	Qualitative—semi-structured interviews were conduted seperatley with each individual. The interviews focused on the following domains: Knowledge of how families can become involved in the veterans mental health care at Oklahoma City VA Medical Centre (VAMC) Perceptions and feelings about the appropriateness of family involvement Benefits that family involvement may have for the veterans, partners and families Concerns about partner participation in the veterans care Logistical issues or barriers to participate in family programming Approaches for inviting partners to participate in family programming (Who? Where? How?)	USA
Temple, J. McInnes Miller, M. Banford Witting, A & Kim, A.B. (2017) [40]	To explore wives’ experiences of living with active duty Marines who are diagnosed with post-traumatic stress disorder.	8 x participants	Qualitative—a phenomenological inquiry utilising semi structured interviews with a central question: What is it like for you to live with an active-duty male marine with PTSD?	USA
Verbosky, S.J. & Ryan, & D.A. (1988) [41]	To explore a) what is the effect of Vietnam veterans’ PTSD symptoms on women partners b) how do the symptoms of PTSD interrelate with identified women’s issues c) is there a relationship between veterans’ symptoms and the coping skill exhibited by women partners	23 x participants	Qualitative—group therapy consisting of structured and non-structured activities. Data was collected from content and process notes from weekly therapy sessions held for significant others. From the sessions the areas that were documented were: Problem identification Effects of PTSD on the subjects Specific women’s issues Available coping skills	USA
Waddell, E Pulvirenti, M & Lawn, S (2016) [42]	To explore the multidimensional nature of experiences for caring for Australian veterans with PTSD from the perspectives of their intimate partners, with an emphasis on understanding coping.	20 x participants	Qualitative—an interpretive phenomenological approach using in-depth semi-structured face-to-face interviews.	Australia
Woods, J. N. (2010) [43]	To explore what is the lived experience of a military dependant spouse when an Army soldier returns from Iraq and/or Afghanistan with Posttraumatic stress disorder.	10 x participants	Qualitative—Phenomenological approach using semi-structured interviews.	USA
Yambo, T.W. Johnson, M, E. Delaney, K. R. & York, J.A. (2016) [44]	To explore the experiences of military spouses living with veterans with combat-related posttraumatic stress disorder.	14 x participants	Qualitative—Husserlian phenomenology using unstructured interviews.	USA
Calhoun, P.S., Beckham, J.C. & Bosworth, H.B. (2002) [45]	To provide further examination of the association between PTSD symptom severity, caregiver burden, and psychological adjustment in partners of patients with PTSD.	71 couples—Vietnam veterans and their partners	Quantitative—survey including demographic data, Zarit Burden interview (22 point self-reporting measure) and Symptom Checklist-90 (SCL-90-R).	USA
Thandi, G., Oram, S., Verey, A., Greenberg, N., & Fear, N.T. (2016) [46]	To fill this gap by investigating the relationship experiences of non-military partners caring for WIS UK military personnel.	25 x participants	Qualitative—semi-structured telephone interviews.	UK
Martinez, L.M (2018) [47]	To examine the relationship between the veteran’s level of disability and dyadic stress on the military caregiver’s overall sense of well-being	70 x participants	Quantitative—Questionnaires used were Demographic questionnaire World Health Organization Disability Assessment Schedule (WHODAS 2.0) Couples Satisfaction Index (CSI-16) Experience in Close Relationships—Short Form (ECR-SF) Multidimensional Scale of Perceived Social Support (MSPSS) Zarit Burden Interview (ZBI) Satisfaction with Life Scale (SWLS) Patient’s Health Questionnaire (PHQ-15)	USA
Murphy, D., Palmer, E., Hill. K., Ashwick, R. & Busuttil, W. (2018) [48]	To explore the experiences and needs of female partners of Veterans with mental health difficulties.	8 x participants	Qualitative—Interpretative phenomenology using semi structured interviews	UK
Waddell, E., Lawn, S., Roberts, L., Henderson. J., Venning, A., Redpath, P. & Sharp-Godwin, T. (2020) [49]	Toexamine the multidimensional nature of experiences of being an intimate partner of a contemporary Veteran with posttraumatic stress disorder (PTSD).	10 x participants	Qualitative—phenomenological approach using face -to-face semi- structured interviews.	Australia
Brickell, T.A., Cotner, B.A., French, L.M., Carlozzi, N.E., O’Connor, D.R., Nakase-Richardson, R. & Lange, R.T. (2021) [50]	To examine the influence of traumatic brain injury (TBI) severity on the health-related quality of life of caregivers providing care to service members/veterans (SMV) following a TBI.	30 x participants	Qualitative—participants attended one of six focus groups	USA
Johnstone, H. & Cogan, N. (2021) [51]	To explore intimate partners’ views of the role they play in supporting Veterans with mental health difficulties and the personal meanings they associate with this role.	6 x participants	Qualitative—phenomenological approach using face-to-face semi structured interviews.	UK

To reduce the risk of bias, all papers included in the review were quality assessed. A process of critical appraisal was executed to determine if the literature was trustworthy, relevant and appropriate to this study [19, 21]. Identifying the strengths and weaknesses in each study allows the researcher the ability to give more weight to stronger papers [22]. The final selection of twenty-seven papers up for scrutiny comprised of eleven quantitative, fourteen qualitative and two mixed methods studies; however, it was the qualitative element within these studies that was of interest for the review. All the quantitative studies selected including mixed methods studies, employed questionnaires/surveys for data collection. An adapted quality assessment tool for quantitative papers was applied to each study [23]. The studies were assessed against seven sections which were ranked as either strong, moderate or weak. An overall ranking was then applied (S2 File Quantitative studies quality ranking). For the remaining sixteen qualitative/mixed methods papers, criteria developed by Kuper, Lingard and Levinson [24] was used to assess domains such as overall coherence of the study, sampling, data collection, analysis, transferability, and ethical considerations. Studies were ranked from unclear, acceptable, good or very good. Papers were included if they ranked acceptable or above in four of the six domains (S3 File: Qualitative studies quality ranking).

## Results

### Paper characteristics

The initial step in identifying thematic groups is to assess the characteristics of the selected studies [18]. The fundamental characteristics within each the twenty-seven studies are identified in Table 3.

**Table 3:** Paper characteristics.

Study ID number	Author	War related				Average relationship(years)	Specific to Spouse only	Couple
		Unspecified	OEF/OIF	OEF/OIF/Vietnam	Vietnam/Persian Gulf			
[25]	Allen et al.		X			4.7		X
[26]	Beckham et al.				X	Not stated		X
[34]	Brown	X				Not stated	X	
[27]	Buchanan et al.		X			12.8		X
[28]	Campbell & Renshaw		X			11.7		X
[35]	Daniels		X			Not stated	X	
[36]	Iniedu	X				Not stated	X	
[29]	Jordan et al.				X	Not stated		X
[37]	Lyons				X	Not stated	X	
[38]	Manguano-Mire et al.	X				Not stated	X	
[39]	Mansfield et al.	X				26.7	X	
[40]	Renshaw & Caska			X—2 STUDY		Not stated	X	
[30]	Riggs et al.				X	Not stated		X
[31]	Sautter et al.				X	Not stated		X
[32]	Sherman et al.				X	Not stated		X
[41]	Temple et al.		X			Not stated	X	
[42]	Verbosky & Ryan				X	US	X	
[43]	Waddell et al.			X		42	X	
[44]	Woods		X			9.3	X	
[45]	Yambo et al.	X				US	X	
[33]	Calhoun et al.				X	Not stated		X
[46]	Thandi et al.		X			Not stated	X	
[47]	Martinez		X			Not stated	X	
[48]	Murphy et al.	X				18.4	X	
[49]	Waddell et al.		X			14.9	X	
[50]	Brickell et al.	X				Not stated	X	
[51]	Johnstone & Cogan	X				Not stated	X	

The focus differed amongst the studies, with nine studies including both veteran and/or serving partner as well as the spouse, denoting the spouse element was part of a much larger study [25–33]. In these cases, the spouse findings have been used within this review. Eighteen studies had specific focus on the spouse [34–51]. Six studies had a specific focus on the spouse experience whilst their partner was still serving [25, 28, 34, 41, 44, 46], the other twenty one studies focused on the spouse experience cohabiting with the veteran population [26, 27, 29–33, 35–40, 42, 43, 45, 47–51]. All but six studies [32, 39, 46–48, 50, 51] paid specific attention to Post-traumatic Stress Disorder (PTSD) and PTSD symptoms only. Within fourteen studies the serving/veteran partner had a clinical PTSD diagnosis [26, 27, 29, 31, 33–39, 42–45]. Seven studies self-reported PTSD with 4 studies [25, 29, 30, 40] using a clinically recognised symptom assessment and classification tool (DSM-III or DSM-IV) to justify participant selection.

Nineteen studies identified specific military conflicts. Nine studies specifically focused on more recent conflicts, Operation Enduring Freedom (OEF) in Afghanistan and Operation Iraqi Freedom (OIF) in Iraq [25, 27, 28, 35, 41, 44, 46, 47, 49] and eight studies related only to the Vietnam or Persian Gulf conflicts [26, 29–33, 37, 42]. One study included participants from both Vietnam and OEF/OIF [43] whilst one study [40] compared and contrasted the findings of two separate studies, where study one focused on OEF/OIF and study two focused on Vietnam. Eight studies did not make any reference to any specific conflict [34, 36, 38, 39, 45, 48, 50, 51].

In all studies included within this review the gender of the spouse was majority female. Nine studies acknowledged the duration of the relationship with six studies identifying an average of up to eighteen years [25, 27, 28, 44, 48, 49] and two studies having an average of twenty-six years plus [39, 43]. Nineteen of the studies did not specify a duration or an average was not calculated [26, 29, 33–36, 38, 45–47, 50, 51].

Across the twenty-seven studies a range of recruitment methods were executed. Ten studies utilised formal avenues; four used couples-based marriage enrich workshops [25, 28, 39, 42], six used outpatient PTSD clinics [26, 31–33, 38, 48], and three via random selection from military records [10, 14, 25]. Six studies used advertising [30, 34, 35, 44, 47, 51], two studies used a snowballing method [11, 23] and two studies utilised a combination of both [41, 42]. Three used third-party services specific to veterans and families [27, 49, 50] and one study recruited from a church group [36].

Twenty-two studies were carried out in the United States of America [25–42, 44, 45, 47, 50]. Two studies were carried out in Australia [21, 27] and three studies were completed in the UK [46, 48, 51]. Two studies utilised a mixed method approach [27, 36], however it is the qualitative element of each study that is relevant for this review, fourteen utilised a qualitative method [32, 34, 37, 39, 41–46, 48–51] and eleven utilised a quantitative method [25, 26, 28–31, 33, 35, 38, 40, 47]. A range of data collection methods were utilised. Thirteen studies carried out questionnaires or surveys [25, 26, 28–31, 33–36, 38, 40, 47] and two studies offered an opportunity for free text within their questionnaire [27, 39]. Seven studies carried out face-to-face interviews [32, 34, 36, 37, 41, 43, 44, 48, 49, 51], one study used telephone interviews [46], and a further study choose to use a combination of face-to-face and telephone interviews [45]. Only one study opted for observation and documentation [42] and one opted for focus groups [50] as the method of choice.

Whilst there was commonality in the overarching themes being tested within the quantitative studies, that unity was not evident in the selection of tools, inventories and scales used to collect the data. Three different scales were used in more than one study. The PTSD checklist (PCL) was utilised in three studies [25, 28, 40] with a further two studies using a military PTSD checklist (PCL-M) [30, 38]. The Burden Inventory was cited in four studies [26, 30, 38, 39] and a further three studies named the Relationship Assessment Scale [28, 40, 47]. All the qualitative studies with the exclusion of one [42] used in-depth semi-structured interviews as the chosen method of data collection [32, 34, 36, 37, 41, 43–46, 48, 49, 51].

Analysis of the retrieved papers was undertaken to identify emerging themes. Five themes were identified; three distinct themes featured in over half of the studies and a further two themes emerged from over 25% of the studies. See Table 4.

**Table 4:** Themes matrix.

Paper number	Theme identified				
	Theme 1 Caregiver burden	Theme 2 Intimate relationship	Theme 3 Psychological /Psychosocial effects on the spouse	Theme 4 Mental health service provision	Theme 5 Spouse’s knowledge and management of symptoms
Allen et al.	X	X			
Beckman et al.	X		X		
Brown	X		X	X	
Buchanan et al.				X	X
Campbell & Renshaw		X			
Daniels	X				
Iniedu		X	X		
Jordan et al.		X	X		
Lyons	X	X	X		
Manguno-Mire et al.	X		X		
Mansfield et al.	X	X		X	
Renshaw & Caska		X			
Riggs et al.		X			
Sautter et al.	X				
Sherman et al.	X	X		X	X
Temple et al.	X		X	X	X
Verbosky & Ryan		X	X		
Waddell et al.	X	X	X	X	
Woods		X	X		
Yambo et al.	X	X	X		X
Calhoun et al.	X		X		
Thandi et al.	X	X	X		X
Martinez		X	X		
Murphy et al.			X		X
Waddell et al.	X	X	X	X	
Brickell at al.	X	X	X	X	
Johnstone & Cogan	X		X		X

The five themes are:

- Theme 1: Caregiver burden
- Theme 2: Relationships
- Theme 3: Psychological/psychosocial effects on the spouse
- Theme 4: Mental health service provision
- Theme 5: Spouse’s knowledge and management of PTSD symptoms.

### Theme 1: Caregiver burden

Caregiver burden is defined as the extent to which caregivers perceive their emotional or physical health, social life, or financial status to be affected by their caring for an impaired relative [52]. The concept of caregiver burden includes both an objective element such as strained relationships, financial constraints, and a subjective element such as the reactions and responses as a result of the demand placed on the carer [53]. Partners of ‘cared for’ individuals are potentially at higher risk of experiencing caregiver burden and poorer mental health as opposed to other family, friend or unrelated carers, due to residing together and increased long-term exposure to each other [54].

The notion of caregiver burden was cited in seventeen studies [25, 26, 31–35, 37–41, 43, 45, 46, 49–51]. Yambo et al. [45] and Sherman et al.’s [32] studies identified two differing types of care burden, first the psychological burden, discussed in theme three and secondly, the burden from the practical and physical actions required from the carer. To provide such support very often required a change in role, which was identified in nine of the studies [32, 34, 37, 39, 41, 43, 45, 46, 49].

Within Brown [34], Lyon [37], Mansfield et al. [39], Temple et al. [41] and Yambo et al.’s [45] studies, the spouses’ stated that they felt more like a care provider than a wife; being an advocate for their serving/veteran partners’ care. A quote from Temple et al. [41] states:

“*the relationship feels like I’m a nurse v’s the spouse*”

(p171).

In some cases, this change of role was taken on voluntarily; however, for some spouses, the change in role felt forced upon them as a result of their serving/veteran partner being unable or unwilling to perform a role within the relationship, [34, 37, 41, 51]. Apparent in four of the studies’ findings [32, 34, 39, 43], was the naivety and the disillusionment of spouses regarding their appreciation of longevity of the caregiver role, thinking that the role would be temporary rather than the emerging long-term/permanent reality which spouses voiced.

One of the ways the long-term impact was identified was the manifestation of the need to constantly maintain the *‘peace’* in order to minimise stress for their serving/veteran partner [39, 43, 45]. Mansfield et al.’s [39] study likened it to

“*walking on eggshells*”

(p492).

Noted across the findings of six studies, was that the physical and mental demands felt from years of providing care, increased stress levels, caused frustration and ultimately, fatigue [26, 34, 39, 43, 49, 50]. The participants in Brickell et al. [50] study highlighted how time-consuming caregiving is and consequently led to exhaustion and being emotionally drained. The burden on the caregiver that this sense of dependency caused was echoed in Waddell et al.’s [49] findings.

In addition, a further attributing factor emerged: there was a significant correlation between caregiver burden and the severity of PTSD symptoms. Similarly, three studies [25, 38, 50], found that serving/veteran partners PTSD severity was a reliable predictor of caregiver burden. As well as exploring the severity of symptoms and caregiver burden, Calhoun et al. [33] study included the level of veteran interpersonal violence; an area not examined in other studies. Findings showed that symptom severity was not solely attributable to caregiver adjustment/burden and that there was a significant association between interpersonal violence and both caregiver burden and partner psychological adjustment.

Seven studies also highlighted a link between the need to be engaged in the serving/veteran partner’s treatment and caregiver burden [32, 35, 38, 43, 49, 50]. Within all seven studies there was a hope and expectation that by being involved with their serving/veteran partner’s treatment plan, their serving/veteran partner’s symptoms would decrease and in turn lessen the overall burden of care they felt.

### Theme 2: Intimate relationships

Central to all people’s lives are relationships. Relationships come in all shapes and sizes, from casual acquaintances to family/blood connections and to intimate relations [55]. The motivation to establish intimacy with others, is part of a basic human need to belong. Intimacy is a complex concept that is multifaceted, with a range of components within it [56]. Explanations of intimate relationships are founded upon research findings from the fields of psychology, neuroscience, sociology, and from family and communication studies [55].

‘Intimate relationship’ was a point of discussion within sixteen of the studies [25, 28–30, 36, 37, 39, 40, 42–46, 48, 49]. Thandi et al.’s study [46] recognised ‘intimacy’ as a key theme divided into two subthemes: physical intimacy and emotional intimacy. Three studies portrayed positive relationship views, all of which were noted to be from the participants’ discussion of either their relationship prior to deployment and/or the onset of PTSD symptoms, or when the spouses’ talked about their commitment to the relationship [28, 34, 37].

Throughout all the studies there was some degree of negative connotation concerning the spouses’ relationship with their serving/veteran partner. Allen et al. [25] Campbell and Renshaw [28], and Renshaw and Caska’s [40] findings suggest that the serving/veteran partner’s recent deployment and subsequent increase in PTSD symptoms was indirectly linked to negative marital functioning but were not statistically significant. Overall, PTSD symptoms and their severity were a specific feature in five of the studies, all of which highlighted a major impact on the marital relationship [29, 30, 36, 39, 50].

One other contributing factor to the impact on the marriage relationship was domestic abuse seen in Jordan et al. [29] and Mansfield et al.’s [39] studies. Mansfield et al. [39] reported that 10.6% of their participants were victims of verbal, emotional or physical abuse. Jordan et al. [29] found the prevalence of abuse by asking for the number of violent acts, including threats of violence over the previous year. For spouses of veterans with PTSD, there was greater incidence of abuse both as victims but also as perpetrators of abuse towards their serving/veteran partner.

Thirteen studies noted changes in personality, difficulties in communication and long-term withdrawal of the serving/veteran partner ultimately leading to emotional numbing or an emotional disconnect [28, 30, 32, 36, 37, 39, 40, 42–44, 46, 49, 50]. Waddell et al.’s [49] study illustrated how intimacy problems surfaced because of participant experiences of emotional alienation from being unable to express or share thoughts and feelings with their serving/veteran partner. Renshaw and Caska [40] suggested the generalised symptoms, such as social withdrawal are easily misinterpreted by spouses as a reflection about them and/or the relationship, whereas physical symptoms were commonly linked to an illness and therefore, posed minimal threats to the relationship. Nevertheless, this distancing and abandonment manifested in most of the studies as frustration with/or sadness, grief and loneliness about the relationship changes.

Thandi et al. [46] found some participants discussed a change in character in their serving partner and that they were no longer like the person they married, which led less affection and more arguments. Whereas, in Waddell et al. [43] findings, participants viewed their relationships as different to others, prompting the notion that their relationship was not a ‘normal’ one. In addition, Waddell et al. [43] and Waddell et al. [49] found that the spouses’ felt there was a constant striving to intimately connect with their serving/veteran partner, again in order to break down the barrier of emotional detachment. Verbosky and Ryan [42] and Thandi et al. [46] found that for some participants the lack of intimacy enhanced the spouse’s need to be nurturing and caring in order to reconnect. Sherman et al. [32] and Waddell et al.’s [43] studies reported participants expressed loyalty and commitment to their serving/veteran partner and described the importance of providing emotional and behavioural support.

This dedication to the relationship was mirrored in Brown [34], Iniedu [36], Lyons [37], Mansfield et al. [39] and Woods [44] findings, although, all reported an ongoing inner struggle as to whether to stay or leave the relationship for most participants. Factors such as children, domestic abuse were listed as reasons to leave; however, these were often overruled by guilt, love, a sense of obligation, and fear that their serving/veteran partner would worsen if they left. Longevity of the relationship was also a consideration which featured in both Allen et al. [25] and Woods [44] studies. Woods’s [44] study showed that those participants with longer relationships were more likely to remain in the marital relationship believing that the relationship was positive whilst those with younger marital relationships, predominantly viewed their relationship, with more negativity. Whereas Thandi et al. [46] and Martinez [47] identified longevity, not by the length of time in the relationship but that a positive relationship was established over a period of time, post diagnosis.

### Theme 3: Psychological/Psychosocial effects on the spouse

Seventeen studies reported on the psychological and psychosocial impact experienced by spouses of either serving personal or veterans with PTSD or other mental health illnesses [26, 28, 29, 33, 36–38, 41–43, 45–51]. Psychological distress was a predominant finding throughout the studies. Manguno-Mire et al. [38], Beckman et al. [26] and Iniedu [36] all indicated that the greater the severity of PTSD symptoms experienced by the serving/veteran partner, a greater intensity of psychological distress, dissatisfaction and anxiety was experienced by the spouse. Manguno-Mire et al. [38] and Brickell et al. [50] studies report high individual measures for anxiety, depressive/somatic symptoms and suicidal ideation with the suggestion that the severity of symptoms might warrant clinical intervention. Iniedu’s [36] study found that all spouses experienced secondary trauma as a result of their serving/veteran partners PTSD symptoms and were in receipt of medication and/or face-to-face therapy. Lyon [37], Waddell et al. [43], Calhoun et al. [33], Murphy et al. [48] and Johnstone and Cogan [51] reported the negative impact that living with a serving/veteran partner with PTSD had on spouses’ mental health by identifying the stress related symptoms they experienced. Manguno-Mire et al.’s [38] study further identified predictors specific to the levels of psychological distress experienced. Psychological distress was found to decrease when there was greater involvement with the serving/veteran partner’s care and treatment; however, if there had been a recent episode of mental health treatment or an increased perceived threat from the serving/veteran partner’s PTSD symptoms, the psychological distress felt by the spouses was also increased. Whereas Martinez [47] examined attachment style and the level of attachment within the relationship and the subsequent effect on psychological and physical symptoms. He found that caregivers with an anxious attachment style were more likely to experience physical symptoms and higher incidents of physiological stress than those with a non-anxious attachment style.

Manguno-Mire et al.’s [38] study identified that 60% of participants reported that their serving/veteran partner posed a physical threat to their wellbeing. The threat and psychological distress were also demonstrated in other studies [41, 46, 47].

Murphy et al. [48] identified the volatile environment where some of the participants likened it to the metaphor:

‘*walking on eggshells*’

(p5);

Albeit a different interpretation of the same metaphor highlighted in the earlier theme. The ‘*not knowing’* and loss of predictability invariably leads to hypervigilance and hyper-attentiveness which was documented in nine studies [28, 33, 37, 43, 45, 48, 49, 51]. Remaining hypervigilant and hyperattentive to the actions and moods of their serving/veteran partner has been aligned with the need to find a resolution and an attempt to create peace and healing [37]. Yambo et al. [43], and Johnstone and Cogan [51] findings suggest an opposing view of an emotionally unstable environment, resulting from the increased feelings of stress from continuous exposure of symptoms, unpredictability and hypervigilance.

As well as spouses being hypervigilant and hyperattentive to their serving/veteran partner’s needs, seven studies [41–43, 46–49] highlight a distinct level of responsibility felt by the spouse. Fear for their serving/veteran partner, guilt linked to the inability of being able to rectify their serving/veteran partner’s difficulties and emotional pain led to feelings of self-hate and blame. Lyon’s [37] study demonstrated the move from the early phases of the relationship, referring to spouses’ feelings being:

‘*compatible with the honeymoon period*’

(p72)

towards the mid phases, where comprehension of the severity of their serving partner’s PTSD and subsequent impact on the relationship are realised. The feelings reported were numerous and varied from happiness and laughter to frustration, resentment and/or bitterness, guilt and humiliation; being out of control and trapped; grief and loss to pride for both themselves and their serving/veteran partners. Temple et al. [41] simply described the relationship as a ‘*roller coaster’*.

This myriad of feelings continues to be identified throughout six studies [34, 36, 42, 48, 50, 51]. The most, negative connotations are described by Brown [34], she points out:

“*anger was used 103 times to describe their feelings in comparison to love at 32 times*”

(p250).

Verbosky and Ryan [42] state that the spouses experienced an overwhelming sense of helplessness and uncertainty as they were unable to formulate plans to effectively deal with the symptoms and situations they faced, finding it difficult to be assertive at the appropriate time. In contrast, Iniedu [36] and Johnstone and Cogan [51] suggest that there was evidence of empowerment brought about by the spouses struggles to cope and hold everything together; indicative of the concept of post-traumatic growth [57].

Amongst the extensive array of feelings identified and the change in behaviours required as a result of the serving/veteran partner’s symptoms, a loss of self was identified in five of the qualitative studies [34, 43, 48, 50, 51]. Brown’s [34] study illustrated that participants had exhausted all their intrinsic resources and faced a lack of normality which in turn meant that many had neglected responsibility for themselves and indeed, lost themselves in a sense of powerlessness.

Whilst Brickell et al.’s [50] study acknowledged the loss of self from an emotive perspective, the loss of physical self-care was also recognised, emerging in their analysis as the most frequently endorsed theme

The transference of loss of self into home and work life was evident in eight of the studies [29, 34, 36, 41–43, 46, 50, 51]. In most, this psychosocial element was identified as “*tremendously stressful*” [34]. Iniedu [36], Temple et al. [41] and Verbosky and Ryan’s [42] studies identified that managing either their serving/veteran partners’ symptoms and/or their own stresses had significant ramifications on daily life and in some cases had taken over completely. Temple et al. [41] and Waddell et al. [43], and Brickell et al.’s [50] studies suggested that the adaptations and modifications required to daily life meant that the spouses had to adjust work hours and, in some cases, reduce hours or quit their job. In addition to the impact on their own lives, there was also the identification of children within such scenarios. Brown [34], Jordan et al. [29] and Temple et al. [41] found similarities in the concern voiced regarding the impact on children and their subsequent behaviours.

It could be suggested that as a result of the negative feelings felt by most spouses within the studies there would be correlation with friendship, socialising and external support (discussed in Theme 4). Nine studies explicitly documented findings highlighting friendship and socialisation [29, 34, 41–43, 47, 48, 50, 51]; Waddell et al.’s [43] study briefly highlighted the spouses’ social isolation whereas Temple et al.’s [41] study explored this in greater depth, finding that the serving/veteran partners’ struggle to leave the house, had an impact on their ability to socialise which led to difficulties maintaining existing friendships or making new ones. Likewise, Brown [34], Verbosky and Ryan [42] and Bickell et al.’s [50] studies indicated that participants merely gave up on any recreational or social activities. Similarly, Murphy et al. [48] and Brickell et al.’s [50] participants felt others (family and friends) who were not living the same experience, simply did not understand. In contrast however, Jordan et al.’s [29] quantitative study found no significant difference in the levels of social isolation.

### Theme 4: Mental health service provision

Mental health service provision emerged as a theme within ten of the papers [27, 32, 34, 39, 41, 43, 46, 48–51] albeit very briefly in three papers [46, 48, 51]. The ability to liaise with medical or other trained professionals with experience of dealing with PTSD was reflective of the spouses perceived individual needs in three studies [34, 39, 48]. Greater involvement in the serving/veteran partner’s care and treatment by the spouse was also noted. Mansfield et al. [37] and Temple et al.’s [41] studies also identified mental health services; namely, requests for help or receiving care. The spouses’ main aims were to gain information in order to inform their care, receive constructive feedback on how they were managing, sharing information that may not have been disclosed by their serving/veteran partner or merely sharing their experiences of daily life. It is evident from the spouses’ experiences however, that these requests were not always received positively by the mental health services. Johnstone and Cogan [51] voiced:

‘*a sense of being invisible, forgotten and overlooked*’

*(p45)*

when it related to their serving/veteran partners’ treatments. Although Murphy et al.’s [48] findings highlighted the value participants felt by being able to share experiences and gain expert in-depth knowledge from specialist practitioners.

Serving/veteran partners had a clinical diagnosis and had in the past or were currently receiving treatment for their illness in sixteen studies. It was also identified in theme three that psychological distress was prevalent throughout seventeen of the studies [26, 29, 33, 34, 36–38, 41–43, 45–51] with six studies [26, 29, 36–38, 43, 51] recognising that the spouse themselves had to seek help or treatment for stress related symptoms. Mansfield et al.’s [39], Waddell et al.’s [43] and Waddell et al.’s [49] studies described similar feelings albeit related to the attempt as seeking help for themselves, feelings of isolation and invisibility were recognised in comments such as:

“*in general family members seem to be left out” and “…..but there is no help for the family*”

(35. p419).

In Buchanan et al. [27], Temple et al. [41], Johnstone and Cogan’s [51] studies, spouses were cautious about reaching out to others since their partners were *serving* military personnel and access to mental health care services differed to that offered to veterans. Buchanan et al.’s [27] study highlighted stigma towards PTSD, which is echoed in the majority of narratives gathered by Temple et al. [41]. In addition to the stigma, the narratives also highlighted the mixed messages received from the military unit. Positive messages surrounding PTSD were promoted through adverts in and around the military base, however, direct actions such as accessing services sent a:

“*negative message that the marine was weak*”

(40. p172)

and reactions received when the serving/veteran partner tried to access services was that:

“*a spouse’s cry for help doesn’t matter*”

(40. p172).

Mirrored in another study, spouses, voiced similar feelings of being:

“*silenced by the institution; by having no voice*”

(27. p240).

A further complication to accessing help and support from mental health services and professionals was the belief that doing so, jeopardised the future career prospects for their serving/veteran partner [27, 39]. One narrative in Temple et al.’s [41] study differed, however; this was from a spouse who was a serving member of the military and whose experience varied as a result of being part of the organisation. For those spouses who did have experience of liaising with services, there were a couple of positive comments raised pertaining to service provision. However, the majority of comments made were critical of the services provided [32, 39, 50].

### Theme 5: Spouse’s knowledge and management of symptoms

Six of the studies highlighted spouse’s knowledge around PTSD and the management of symptoms when they occurred [27, 32, 41, 45, 46, 48]. Buchanan et al.’s [27] study specifically focused on the awareness of PTSD from the spouse perspective. They undertook a critical incident survey which included the question *“How would you know if your spouse/partner needed treatment for PTSD*?*”* The findings suggested that two thirds of spouses had received no formal training on PTSD and most spouses had accessed informal sources to learn about PTSD. Media resources such as movies, news broadcasts or internet were identified as primary sources. Murphy et al.’s [48] study suggested that as a result of a sense of responsibility, practical learning about what to do and say was valued by the participants. Temple et al.’s [41] study presented one spouse who differed from the other spouses; she voiced a clear understanding and underpinning knowledge of PTSD symptomology which she attributed to the in-house training she had received as a serving member herself.

Buchanan et al.’s [27] study explored further spouses’ knowledge and understanding about PTSD causes, a fifth of spouses were able to identify the causes relating to their serving/veteran partners. 12% of participants declared they had little knowledge of the presenting symptoms [27]. While Murphy et al.’s [48] study didn’t specifically explore an individual’s knowledge, it highlighted the need to share experiences with peers in similar situations in order to gain reassurance and increase confidence in their understanding.

One of the key themes emerging from Sherman et al. [32], Temple et al. [41], Yambo et al. [45] and Thandi et al.’s [46] studies into spouses’ experience of living with serving/veteran partners with PTSD, was being unprepared to handle the condition and/or deal with the complexity of the symptoms. Thandi et al.’s [46] participants.

‘*described how they felt ill-equipped to perform the role as caregiver*’

(p2).

Most participants in Temple et al. [41] and Yambo et al.’s [45] studies stated they had never been provided with any information about PTSD either before or after their serving/veteran partners’ deployment and consequently were unable to identify whether their serving/veteran partners had PTSD. As a result, of the lack of information around PTSD, spouses begun to doubt their relationship and own sanity and believed that they were to blame for their serving/veteran partners’ destructive behaviours; and for some spouses, this belief had exceeded 10 years.

## Discussion

Following completion of the review, it was apparent that there was a limited range of papers where the primary focus was the experience of the spouses of serving military personnel. As explained earlier the parameters of the search had to be widened to include spouses of veterans and the time scale was broadened to include studies undertaken post the Vietnam conflict. On reviewing the available literature, five predominant themes emerged. Interlinked themes were identified it was sometimes difficult to separate findings into one distinct theme since in most cases, they often interlinked.

The notion of caregiving burden was evident in several papers. Within most studies’, caregiver burden was viewed negatively. Evident in the literature was how the spouses’ level of burden increased at times when their serving/veteran partners’ symptoms of PTSD were at their most severe. Likewise, when their serving/veteran partners’ PTSD symptoms were minimal and they were responding well to an aspect of treatment, the level of caregiver burden felt by the spouses lessened.

As well as the perceived caregiver burden, the impact on the relationship was also apparent and emerged as another key theme. The majority of spouses were married and had been a part of military life whilst their serving/veteran partners were serving in the case of the veterans. The toll on the relationship was evident, with many spouses stating that they had—at times—felt like leaving the relationship. Many spouses blamed themselves for the problems faced in the relationship. In some of the literature, accounts about the relationship prior to their serving/veteran partners’ PTSD illness were taken from the spouses. These were reflected on with fondness and love akin to the ‘honeymoon period’. Once symptoms such as emotional detachment entered the relationship, the relationship became much harder, and problems began to escalate. Many spouses felt a sense of responsibility to stay and ‘*stand by their man’*, and in all but one of the papers the spouses had stayed. Some of this was out of fear that their serving/veteran partner would hurt themselves or become worse. For some, it was out of loyalty, for some it was guilt about deserting them in their time of need and for some it was love. Very often it was mixture of all these reasons, meaning the relationship was no longer viewed as ‘normal’.

The decision to stay had ramifications psychologically and psychosocially on the spouse. Throughout many of the studies, it was evident that they found coping with everything- family, home, work and their serving/veteran partner -stressful and anxiety provoking. This stress led to many spouses seeking treatment for their own mental health needs. Spouses described being peacekeepers to prevent triggering their serving/veteran partners’ symptoms. Spouses became hyper-vigilant and hyper-attentive to their serving/veteran partners’ behaviours and needs which, ultimately, placed greater strain on themselves. Spouses also described how their lives had changed socially; some felt forced to reduce their working hours, withdrawal from maintaining existing friendships and/or making new acquaintances due to caring for their serving/veteran partners’. For most, the spouse experiences held negative connotations with few studies exploring resilience, growth and/or transformation of self or the relationship.

A small number of studies explored what knowledge and insight spouses held about PTSD or mental health issues. For the majority, no formal training or guidance had been received and most of the spouses had used media such as films, internet, and campaigns to make the connection between their serving/veteran partners’ symptoms and mental health issues. Mixed messages were also highlighted; however, this was predominantly from those studies where the serving/veteran partner was still serving. For these spouses, there was an element of fear about upsetting the ‘*applecart*’; they were frightened that disclosing their serving/veteran partners’ symptomology or seeking help would affect their serving/veteran partners’ career prospects. Further, that their serving/veteran partners would be stigmatised by a diagnosis despite widespread use of flyers and advertisements stating that it was ‘*ok to talk’*. Spouses felt torn between the need to help their serving/veteran partner verses jeopardising their partners’ career. Many spouses felt invisible and isolated with nowhere to turn for support for either their serving/veteran partners or themselves.

Barriers to mental health service provision were also recognised; for some it was the financial burden, for others accessibility and/or time and/or not even knowing where to go in the first instance. For those who had accessed mental health services, the experience was far from ideal for most; staff shortages, lack of funding, long waiting times and poor facilities meant disappointment once access was finally gained.

## Limitations to current research and systematic review and narrative synthesis

Employing a systematic search strategy ensured that the searches were transparent. Despite adopting the systematic approach, only a limited number of contemporary papers specific to the military spouses were yielded. The lack of peer reviewed studies over recent years internationally, provided the rationale for the inclusion of earlier studies. These were identified by increasing the time parameters and by executing a reference and citation search on the papers found; again, this yielded only a few earlier papers for inclusion. From the twenty-seven studies identified, nineteen of the papers focused primarily on the spouses’ experiences. However, only a few specifically pertained to the spouse experiences of serving personnel; the majority were spouses of veterans.

Owing to the cultural complexities across military organisations, studies conducted with westernised military spouses published in peer reviewed English language journals were deemed appropriate to expand this review. A major limitation is the distinct lack of studies carried out outside of the USA; only five studies identified in the UK or Australia. Whilst all the studies used were from westernised cultures, the differences in deployment terms, and healthcare systems are noteworthy. This would make the transferability of some of the findings across countries problematic.

A further limitation surrounded the specifics of the mental health issue itself. The emphasis in most of the included papers were specific to either experience of service personnel directly after deployment with PTSD, or veterans who were no longer serving with PTSD. PTSD was the single focus for many of the papers; only six papers referred to other mental health illness as well as PTSD. Many of the papers in the review made specific links to war as a precursor to the PTSD. The papers gathered made links to either post service in the Vietnam conflict or after serving OIF & OEF conflicts. It is noteworthy that the conflicts were fought 25 years apart and also in different countries and terrains. They were fought by very different means, in that Vietnam used predominantly guerrilla warfare tactics with a largely unseen enemy, whereas OIF and OEF were more conventional in the type of warfare deployed; for example, soldiers faced a modern military organisation with greater use of armoured and air support. These differences suggest that the experiences and exposure faced by those serving, could have been markedly different.

This review is focused specifically on spouses to military personnel or veterans who have served and therefore is not inclusive of the wider literature exploring those spouses’ experience outside of a military context. This focus was intentional, due to the differences in mental healthcare provision for serving personnel. When considering non- military civilian couples, the majority have, and will access the same healthcare provision/organisation. This has advantages such as information sharing between professionals for the provision of holistic family care. Whereas, with most residing military couples, the serving military member accesses different care provision to their family. Accessing separate care provisions provides a potential barrier to information sharing and access to support.

It is widely acknowledged that there are a range of programmes/interventions that aim to offer support to spouses who find themselves experiencing life alongside a serving/veteran partner who has a mental health issue; for example, Spencer-Harper et al.’s [58] study of group psychoeducation support. As a result of such programmes/interventions, it is understood that grey literature exists by wider professional, charitable organisation and government publications. Only peer reviewed research was included in this review which meant that all grey literature was excluded. Two further exclusions were domestic violence and secondary PTSD. This was purposeful, as the aim of this review was to explore the experiences of spouses and not the outcome resulting from the experience. It is widely acknowledged, that the potential outcomes of living with someone with PTSD, are, a higher incidence of intimate partner violence [59] and a higher incidence of secondary PTSD for the spouse [60]. It was felt that the inclusion of such studies would detract from and overshadow the limited peer reviewed literature available.

## Conclusion

The review has identified that there remains a gap in the literature, specifically, studies focusing on military spouses of serving personnel; most of the studies focused on spouses of veterans, but similarities were noted. The majority of the papers reside in the USA (n = 22), with minimal papers from the UK and Australia (n = 3 and n = 2 respectively). While there was a near equal divide between quantitative or mixed methods and qualitative [n = 11+n = 2 and n = 14], only nine studies used interviews as the data collection method. Thus, posing a further limitation as the majority of data collected, lacked the rich, in-depth nature required to explore spouse experience.

The findings from the review have some implications for policy, practice and research focusing on the military spouses’ experiences of living alongside their serving/veteran partners during a mental health issue. Care burden from both a psychological and a physical/practical aspect was evident, as was the longevity of their partners’ mental health issues. All led to long-term impact, where for most military spouses felt more like care providers than partners. The impact was also felt in the intimate relationship between military spouse and partner; difficulties in communication and emotional numbing were identified. However, dedication and commitment to the relationship was also noted. For the military spouses’ themselves, there was a sense of ‘*loss of self’* as a direct result of caring for their partner. In addition, there was a felt sense of being invisible and/or overlooked by the mental health services; when all that was required was inclusion to gain information, so that they could better manage their partners’ care. Understanding the experiences, perspectives and difficulties of military spouses whilst living alongside their serving partner/veteran during a mental health issue, will assist in better understanding of how their interactions can support or implicate their partners’ recovery. Inclusion from services needs to be considered as a protective factor for both the military spouse and their serving partner.

## Supporting information

S1 File PRISMA checklist. (DOCX)

S2 File Quantitative studies quality ranking: Quality assessment tool developed by Thomas et al. [21]. (DOCX)

S3 File Qualitative studies quality ranking: Quality assessment using Kuper, Lingard and Levinson guidelines. (DOCX)
