# Supplementary material for: The military spouse experience of living alongside their serving/veteran partner with a mental health issue: A systematic review and narrative synthesis
Source: PLoS One. 2023 May 18;18(5):e0285714. doi: 10.1371/journal.pone.0285714 (PMC10194995; doi:10.1371/journal.pone.0285714)
Supplement: S2 File — (DOCX) [file pone.0285714.s002.docx]

*Pages correspond to version submitted to journal.

S2 Fig. Quantitative studies quality ranking: Quality Assessment Tool developed by Thomas et al [20]

Note: Each section has individual component rating which is cross referenced against followed by an overall rating as Strong (1), Moderate (2) Weak (3)

|  | Allen et al | Beckman et al | Daniels | Campbell &  Renshaw | Jordan  et al | Manguno-Mire et al | Renshaw &  Caska | Riggs et al | Sautter  et al | Calhoun et al | Martinez |
| --- | --- | --- | --- | --- | --- | --- | --- | --- | --- | --- | --- |
| 1. **Selection bias**   **A** Are the individuals selected to participate in the study likely to be representative of the target population?  **B** What percentage of selected individuals agreed to participate? | 1  1 | 1  N/A | 1  3 | 1  3 | 1  1 | 1  3 | 1  5 | 1  N/A | 1  1 | 1  5 | 1  2 |
| **Section rating** | **1** | **2** | **3** | **3** | **1** | **3** | **2** | **2** | **1** | **2** | **2** |
| 1. **Study design**   **D** Was the study described as randomized? If NO, go to section 3. | 4 | 5 | 7 | 7 | 4 | 7 | 3 | 8 | 8 | 8 | 4 |
| **Section rating** | **2** | **2** | **2** | **2** | **2** | **2** | **2** | **3** | **3** | **2** | **2** |
| 1. **Confounders**   **E** Were there important differences between groups prior to the intervention?  **F** If yes, indicate the percentage of relevant confounders that were controlled (either in the design (e.g.  stratification, matching) or analysis)? | 1  1 | N/A  N/A | N/A  N/A | N/A  N/A | 1  3 | N/A  N/A | 2  N/A | 1  1 | N/A  N/A | 1  1 | N/A  N/A |
| **Section rating** | **1** | **-** | **-** | **-** | **3** | **-** | **2** | **1** | **-** | **1** | **-** |
| 1. **Blinding**   **G** Was (were) the outcome assessor(s) aware of the intervention or exposure status of participants?  **H** Were the study participants aware of the research question? | N/A  N/A | 1  3 | N/A  N/A | N/A  N/A | N/A  N/A | N/A  N/A | N/A  N/A | 1  1 | N/A  N/A | 1  3 | N/A  N/A |
| **Section rating** | **-** | **3** | **-** | **-** | **-** | **-** | **-** | **1** | **-** | **3** | **-** |
| 1. **Data collection methods**   **I** Were data collection tools shown to be valid?  **J** Were data collection tools shown to be reliable? | 1  1 | 1  1 | 1  1 | 1  1 | 1  1 | 1  1 | 1  1 | 1  1 | 1  1 | 1  1 | 1  1 |
| **Section rating** | **1** | **1** | **1** | **1** | **1** | **1** | **1** | **1** | **1** | **1** | **1** |
| 1. **Withdrawals and drop-outs**   **K** Were withdrawals and drop-outs reported in terms of numbers and/or reasons per group?  **L** Indicate the percentage of participants completing the study. (If the percentage differs by groups, record the  lowest). | 3  4 | 3  4 | 1  1 | N/A  N/A | N/A  N/A | 1  4 | N/A  N/A | N/A  N/A | 1  1 | 4  1 | N/A  N/A |
| **Section rating** | **3** | **3** | **1** | **-** | **-** | **3** |  | **-** | **1** | **2** | **-** |
| 1. **Analyses**   **M** Indicate the unit of analysis :community(1) organization/institution(2) practice/office(3) individual(4)  **N** Are the statistical methods appropriate for the study design**?** | 2  1 | 2  1 | 4  1 | 2  1 | 2  1 | 2  1 | 2  1 | 2  1 | 2  1 | 2  1 | 4  1 |
| **Section rating** | **1** | **1** | **2** | **1** | **1** | **1** | **1** | **1** | **1** | **1** | **2** |
| **Overall rating** | **2**  **Moderate** | **2**  **Moderate** | **2**  **Moderate** | **2**  **Moderate** | **2**  **Moderate** | **3**  **Weak** | **1**  **Strong** | **2**  **Moderate** | **2**  **Moderate** | **2**  **Moderate** | **2**  **Moderate** |
