# Supplementary material for: The military spouse experience of living alongside their serving/veteran partner with a mental health issue: A systematic review and narrative synthesis
Source: PLoS One. 2023 May 18;18(5):e0285714. doi: 10.1371/journal.pone.0285714 (PMC10194995; doi:10.1371/journal.pone.0285714)
Supplement: S3 File — (DOCX) [file pone.0285714.s003.docx]

S3 Fig. Qualitative studies quality ranking: Quality assessment using Kuper, Lingard and Levinson guidelines.

*Note. (1) VG: Very Good; G: Good; A: Acceptable; U: Unsure; (2) Inclusion in review: Studies were required to meet four of six guidelines and be ranked ‘acceptable’ or above.*

| Source | Data analysis method | Was it clear what the researcher did? | Was the sample appropriate? | Was the data collected appropriately? | Was the way the data was analysed appropriate? | Are the results transferable? | Was ethics considered? |
| --- | --- | --- | --- | --- | --- | --- | --- |
| Brown, V. A. (2015) [25] | Thematic analysis | VG | G | VG | VG | G | VG |
| Buchanan, C., Kemppaninen, J., Smith, S., MacKain, S. & Wilson-Cox, C. (2011) [26] | Thematic analysis | VG | VG | G | VG | G | G |
| Iniedu, A.O.E. (2010) [29] | Thematic analysis | VG | G |  | G | G | VG |
| Lyons, M. A. (1999) [31] | Thematic analysis | G | G | VG | G | G | VG |
| Mansfield, A.J., Schaper, K.M., Yanagida, A.M. & Rosen, C.S. (2014) [33] | Thematic analysis | G | VG | A | A | A | A |
| Sherman, M.D. Blevin, D. Kirchner, J Ridner, L & Jackson, T (2008) [37] | Thematic analysis | G | G | VG | G | G | U |
| Temple, J. McInnes Miller, M. Banford Witting, A & Kim, A.B. (2017) [38] | Thematic analysis | A | A | G | G | G | A |
| Verbosky, S.J. & Ryan, & D.A. (1988) [39] | Thematic analysis | A | VG | A | A | A | U |
| Waddell, E Pulvirenti, M & Lawn, S (2016) [40] | Thematic analysis | G | VG | G | VG | G | VG |
| Woods, J. N. (2010) [41] | Thematic analysis | G | G | VG | VG | G | VG |
| Yambo, T.W. Johnson, M, E. Delaney, K. R. & York, J.A. (2016) [42] | Thematic analysis | G | G | G | VG | G | VG |
| Thandi, G., Oram, S., Verey, A., Greenberg, N., & Fear, N.T. (2016) [44] | Thematic analysis | G | VG | G | VG | G | VG |
| Murphy, D., Palmer, E., Hill. K., Ashwick, R. & Busuttil, W. (2018) [46] | Thematic analysis | G | G | VG | VG | G | VG |
| Waddell, E., Lawn, S., Roberts, L., Henderson. J., Venning, A., Redpath, P. & Sharp-Godwin, T. (2020) [47] | Thematic analysis | G | G | VG | VG | G | VG |
| Brickell, T.A., Cotner, B.A., French, L.M., Carlozzi, N.E., O’Connor, D.R., Nakase-Richardson, R. & Lange, R.T. (2021) [48] | Thematic analysis | VG | VG | G | VG | G | VG |
| Johnstone H. & Cogan, N. (2021) [49] | Thematic analysis | G | A | G | VG | A | VG |
